# Pre-Existing Dengue Immunity Drives a DENV-Biased Plasmablast Response in ZIKV-Infected Patient

**DOI:** 10.3390/v11010019

**Published:** 2018-12-29

**Authors:** Siddhartha K. Bhaumik, Lalita Priyamvada, Robert C. Kauffman, Lilin Lai, Muktha S. Natrajan, Alice Cho, Nadine Rouphael, Mehul S. Suthar, Mark J. Mulligan, Jens Wrammert

**Affiliations:** 1 Division of Infectious Disease, Department of Pediatrics, Emory University School of Medicine, Atlanta, GA 30322, USA; siddharthabhaumik@gmail.com (S.K.B.); odk7@cdc.gov (L.P.); robert.kauffman@emory.edu (R.C.K.); alice.hong.cho@emory.edu (A.C.); mehul.s.suthar@emory.edu (M.S.S.); 2 Emory Vaccine Center, Emory University School of Medicine, Atlanta, GA 30322, USA; 3 Hope Clinic of the Emory Vaccine Center, Division of Infectious Diseases, Department of Medicine, School of Medicine, Emory University, Decatur, GA 30033, USA; llai@emory.edu (L.L.); muktha.natrajan@emory.edu (M.S.N.); nroupha@emory.edu (N.R.); mark.mulligan@nyulangone.org (M.J.M.)

**Keywords:** ZIKV, DENV, antibodies, plasmablast, B-cell, cross-reactivity

## Abstract

The re-emergence of Zika virus (ZIKV) in the western hemisphere has most significantly affected dengue virus (DENV) endemic regions. Due to the geographical overlap between these two closely related flaviviruses, numerous individuals who suffered ZIKV infection during recent outbreaks may have also previously been exposed to DENV. As such, the impact of pre-existing dengue immunity on immune responses to ZIKV has been an area of focused research and interest. To understand how B cell responses to a ZIKV infection may be modulated by prior dengue exposures, we compared and contrasted plasmablast repertoire and specificity between two ZIKV-infected individuals, one dengue-naïve (ZK018) and the other dengue-experienced (ZK016). In addition to examining serological responses, we generated 59 patient plasmablast-derived monoclonal antibodies (mAbs) to define the heterogeneity of the early B cell response to ZIKV. Both donors experienced robust ZIKV-induced plasmablast expansions early after infection, with comparable mutational frequencies in their antibody variable genes. However, notable differences were observed in plasmablast clonality and functional reactivity. Plasmablasts from the dengue-experienced donor ZK016 included cells with shared clonal origin, while ZK018 mAbs were entirely clonally unrelated. Both at the mAb and plasma level, ZK016 antibodies displayed extensive cross-reactivity to DENV1-4, and preferentially neutralized DENV compared to ZIKV. In contrast, the neutralization activity of ZK018 mAbs was primarily directed towards ZIKV, and fewer mAbs from this donor were cross-reactive, with the cross-reactive phenotype largely limited to fusion loop-specific mAbs. ZK016 antibodies caused greater enhancement of DENV2 infection of FcRγ-expressing cells overall compared to ZK018, with a striking difference at the plasma level. Taken together, these data strongly suggest that the breadth and protective capacity of the initial antibody responses after ZIKV infection may depend on the dengue immune status of the individual. These findings have implications for vaccine design, given the likelihood that future epidemics will involve both dengue-experienced and naïve populations.

## 1. Introduction

Zika virus (ZIKV) was one of several lesser-known and understudied flaviviruses until about a decade ago when it caused a burst of infections in the Pacific Islands [1,2] and more recently, widespread epidemics in South and Central America and the Caribbean [3,4]. The recent outbreaks saw a high incidence of ZIKV-related Guillain–Barré syndrome in adults [5] and Congenital Zika Syndrome in fetuses born to ZIKV-infected pregnant mothers [6,7,8], which had not been reported in previous ZIKV outbreaks [9,10,11]. High sequence and structural homology between dengue virus (DENV) and ZIKV [12,13] leading to similar immunological epitopes, as well as the co-circulation of these flaviviruses [14,15], have made the serology-based diagnosis of ZIKV and the development of novel vaccines that can protect against both viruses a serious challenge [16,17,18].

In the past few years, a number of studies have demonstrated immunological cross-reactivity between DENV and ZIKV, showing cross-neutralization of ZIKV by DENV antibodies and vice-versa [13,19,20,21]. By testing human serum/plasma or monoclonal antibodies (mAbs) generated from single B cell clones, these studies have shown that the envelope protein (E) is the main target for cross-protective antibodies both in vitro and in vivo [13,19,22,23]. Consequently, several potent broadly neutralizing mAbs have been identified and evaluated for prophylaxis and/or therapy [19,24,25], and vaccine constructs involving ZIKV E are being developed for clinical trials [26,27]. What complicates matters, however, is the potential for cross-reactive antibodies to cause antibody-dependent enhancement (ADE), a mechanism through which sub-neutralizing concentrations of pre-existing cross-reactive antibodies enhance viral uptake and infection of Fc gamma receptor (FcγR)-bearing cells [28]. Though its physiological relevance for human disease in the context of ZIKV infections is contentious, ADE remains a concern due to the vast global distribution and disease burden of DENV today [29,30].

At present, there is a limited understanding of the kinetics and functional quality of antibody responses generated immediately following ZIKV infection, and even lesser information about how these factors are modulated by prior flavivirus exposures. To address this gap in knowledge, a few groups have delved into to the very early B cell response in ZIKV patients [21,31,32]. These studies have shown that ZIKV-specific antibodies appear in circulation within a few days post symptom onset (DPO) [21,31,32]. These antibodies are the result of the expansion of acute-phase B cells known as plasmablasts, which appear in the periphery transiently following infection [31]. Plasmablasts of IgM, IgA and IgG isotypes have all been shown to contribute to the acute ZIKV humoral response [31]. As early as a week after symptom onset, plasmablasts represented nearly two-thirds of the entire peripheral B cell population (63%) in a dengue-experienced donor in a study by Rogers et al. [21]. This group observed that the magnitude of the plasmablast response varied amongst donors and appeared to differ based on the DENV-exposure status of the individual; dengue-experienced donors generated 15–25 fold larger plasmablast expansions compared to the small plasmablast population seen in dengue-naïve donor in their study. However, flavivirus-naïve donors showed substantial plasmablast responses early after infection in studies by Lai et al. and Ricciardi et al. [31,32]. Additionally, while minimal ZIKV-binding and neutralizing plasma titers were reported for the dengue-inexperienced donor by Rogers et al., both Lai et al. and Ricciardi et al. showed considerable binding and neutralizing plasma titers in their three dengue-naïve patients [21,31,32]. Given these disparate observations, additional studies involving donors with varied dengue serostatus could improve our understanding of the vast inter-donor diversity of the ZIKV B cell response.

Here, we build on existing literature by examining the plasma and plasmablast response in two ZIKV-infected individuals: one with strong serological evidence of prior dengue infection, and the other with minimal serological cross-neutralization of DENV, presumptively without earlier dengue exposures. We generated a panel of 59 mAbs from single-cell sorted patient plasmablasts to analyze the cellular repertoire and the functional quality of the early phase B cell response to ZIKV. A vast majority of the mAbs from the dengue-experienced donor were cross-reactive and neutralized DENV, and plasma neutralizing titers to DENV were higher than to ZIKV, altogether suggesting a DENV-biased recall response. At sub-neutralization concentrations, plasma and mAbs from this patient also potently enhanced DENV2 infection in vitro. In contrast, the DENV-inexperienced patient showed high plasma neutralizing titers to ZIKV but poorly neutralized DENV. The mAbs from this donor also preferentially neutralized ZIKV and were clonally unrelated, resembling a primary response. Interestingly, both the DENV-exposed and naive donors had comparably-sized plasmablast expansions with similar levels of somatic hypermutations in antibody variable genes. Regardless, the breadth and potency of DENV-ZIKV cross-reactivity differed greatly between the two donors. The differences in the antibody repertoire and functional characteristics between the two patients reported in this study are striking, and indicate that prior dengue exposures play an important role in shaping immune responses to future ZIKV infections.

## 2. Materials and Methods

### 2.1. Patients

Patients ZK016 and ZK018 were selected from a cohort of ZIKV patients enrolled at the Hope Clinic of the Emory Vaccine Center in an Emory University IRB approved study of emerging infectious diseases (IRB00022371; 2016–2019). Whole blood, PBMCs and plasma samples were collected and provided to our laboratory for experimentation and analysis.

### 2.2. Viruses, Viral Antigens and Recombinant Proteins

The virus strains used in this study were ZIKV PRVABC59, DENV1 WP, DENV2 Tonga/74, DENV3 Sleman/78 and DENV4 Dominica/84. All viral stocks were passaged in Vero cells as previously described [13,33], titered and stored at −80 °C. The ZIKV lysate was prepared as described previously [13] and the protein concentration was quantified using Bradford reagent. The ZIKV recombinant E and NS1 proteins were purchased from Meridian Life Science (Memphis, TN, USA; R01635, R01636). The DENV recombinant E and NS1 proteins were purchased from CTK Biotech (San Diego, CA, USA; A2301 DENV1-E, A2302 DENV2-E, A2303 DENV3-E, A2304 DENV4-E, A2311 DENV1-NS1, A2312 DENV2-NS1, A2313 DENV3-NS1, and A2314 DENV4-NS1).

### 2.3. ELISpot Assay

The frequency of ZIKV-specific antibody secreting cells (ASCs) was determined by ELISpot assay as previously described [31]. To determine the ZIKV-specific ASC response, 96-well ELISpot filter plates (MilliporeSigma; Burlington, MA, USA; MSHAN4B50) were coated with 10 µg/well of either ZIKV E or NS1. To capture the total IgG ASC response, wells were coated with 5 µg/mL of donkey anti-human IgG antibody (Jackson ImmunoResearch; West Grove, PA, USA; 709-005-149).

### 2.4. Flow Cytometry and Single-Cell Sorting of Plasmablasts

PBMCs obtained from the two patients were stained with titrated amounts of CD19-FITC (BD; clone HIB19), CD3-Pacific Blue (BD; clone SP34-2), CD20-PECy7 (BD; clone L27), CD27-APC (eBiosciences; clone O323) and CD38-PE (BD; clone HIT2). The plasmablast population was defined as CD3− CD19+ CD20−/low CD27+ CD38+ lymphocytes and its frequency analyzed using FlowJo software. The single-cell sorting of plasmablasts was carried out using the FACSAriaII sorter (Becton Dickinson; Franklin Lakes, NJ, USA) at the Emory University Pediatrics Flow Cytometry Core Facility under negative air pressure. The cells were sorted into a 96-well PCR plate as described in [34,35], rapidly frozen on dry ice and stored at −80 °C for subsequent cDNA synthesis.

### 2.5. Monoclonal Antibody Production

The mAbs were produced from the single-cell sorted plasmablasts as described [34,36], with a few modifications at the reverse transcription step. The single-cell sorted plates were thawed on ice and Triton X-100 (Sigma-Aldrich; St. Louis, MO, USA; T8787-50ML) was added at a final concentration of 0.2% to lyse cells. The synthesis of cDNA was performed using 1 µM gene specific anti-sense primer cocktail (heavy, kappa and lambda) and Sensiscript reverse transcriptase (Qiagen; Venlo, Netherlands; 205211). Ig heavy chain and light chain (kappa and lambda) variable domain were amplified by nested PCR using RT-PCR primers listed previously [34]. For sequencing of the heavy and light chain variable domains, another round of nested PCR was performed with an added 5′ M13R sequence (5′-AACAGCTATGACCATG-3′) to each sense primers. Both PCRs were performed using Hot Start Taq Plus Master Mix (Qiagen; Venlo, Netherlands; 203643) with 200 nM primer concentration. The cloning PCR was performed using Phusion Hot Start II DNA Polymerase (NEB; Ipswich, MA, USA; F-549) and V and J gene family specific primers containing appropriate restriction sites as described [34]. To protect the variable domain that has internal restriction sites from being truncated, the cloning PCR was modified using 50 µM 5-methyl-dCTP (NEB; Ipswich, MA, USA; N0356S) and 150 µM dCTP. The heavy chain variable domains, irrespective of the original isotype, were cloned into human IgG1 heavy chain expression vector, while the light chain variable domains were cloned into their respective human light chain (kappa or lambda) expression vector [34]. These vectors were then co-transfected into Expi293F cells according to the manufacturer’s protocol (Thermo Fisher Scientific; Waltham, MA, USA; A14527). Antibodies generated in the cell culture supernatants were purified using Protein A agarose beads (Pierce; Waltham, MA, USA 20333).

### 2.6. Ig Variable Chain Repertoire and Clonality Analysis

The variable region of heavy chain (VH) and light chain (VL) were analyzed for germline variable domain gene usage, complementarity determining region 3 (CDR3) lengths and somatic hypermutation (SHM) using ImMunoGeneTics (IMGT) database and IgBLAST tool. SHM frequencies in the VH and VL sequences were analyzed by matching their sequence to the closest germline sequence and determining the total number of silent (S) and replacement (R) mutations from the framework region 1 (FR1) till before the CDR3. The CDR3 mutations were not included in the analysis as the junction region of the reference germline sequences were estimated with low confidence. Clonality analysis was performed by sequence alignment of the rearranged VH and VL chains with matching V and J gene usage with identical junctional diversity.

### 2.7. ELISA

ZIKV-specific IgM antibodies in patient and healthy donor plasma were measured by the Zika IgM Antibody Capture Enzyme-Linked Immunosorbent Assay (Zika MAC-ELISA) as described [37,38]. To detect ZIKV-specific IgG antibodies, the Zika MAC-ELISA was adapted from published methods [39] with slight modifications: plates were coated with anti-human IgG (KPL Seracare; Milford, MA, USA; 01-10-06) at a dilution of 1:500, followed by subsequent steps as described [37,38].

To determine plasma/mAb binding to ZIKV lysate or ZIKV/DENV E and NS1 proteins, Nunc Maxisorp ELISA plates (eBioscience; Waltham, MA, USA; 44-2404) were coated with the appropriate antigen diluted in PBS overnight at 4 °C: 50 µg/mL of ZIKV lysate, or 1 µg/mL recombinant protein respectively. Plates were then washed with PBS containing 0.05% Tween-20 (PBS-T) and blocked with PBS-T containing 10% FBS (PBS-T-FBS) for 1.5 h. Serially diluted plasma in PBS-T-FBS was added to the plates for 1.5 h. Plates were washed with PBS-T and peroxidase-conjugated anti-human IgG (Jackson ImmunoResearch; West Grove, PA, USA; 109-036-098) was added for 1.5 h. The plates were then washed with PBS-T followed by PBS and developed using an o-phenylenediamine substrate (Sigma; Burlington, MA, USA; P8787) for absorbance detection at 490 nm. The whole virus capture ELISA for ZIKV and DENV was performed as described [13]. The pan-flavivirus 4G2 antibody used to capture virions was generated using the D1-4G2-4-15 hybridoma (ATCC; Manassas, VA, USA; HB-112).

### 2.8. Antibody Competition ELISA

ZIKV E-specific mAbs that target the conserved FL domain in the ZIKV E protein were determined by competing them against FL-specific mouse mAbs 4G2 (ATCC; Manassas, VA, USA HB-112) [40] and E18 (BEI Resources; Manassas, VA, USA; NR-10135) [41] using an ELISA-based assay. ELISA plates were coated with 1 µg/mL of ZIKV E protein diluted in PBS overnight at 4 °C. The plates were washed and blocked as described above. E18 and 4G2 were incubated with either ZIKV E-specific mAbs (competitor mAb) or with PBS-T-FBS and added to the ELISA plate. After 1.5 h, the plates were washed and peroxidase-conjugated anti-mouse IgG (H + L) (KPL Seracare; Milford, MA, USA; 074-1806) was added. Later, the plates were developed with o-phenylenediamine substrate. Human mAbs that caused more than 50% reduction in the binding of mouse mAbs 4G2 and E18 to the ZIKV E protein compared to the no competition control were identified as FL-specific.

### 2.9. Focus Reduction Neutralization Test

The virus neutralization abilities of patient plasma (heat inactivated) and mAbs were determined by the focus reduction neutralization test (FRNT) as previously described [33]. Serially diluted plasma or mAbs were incubated with 60–100 focus forming units of ZIKV, DENV1, DENV2, DENV3 or DENV4 for 1 h at 37 °C. The virus-antibody mixture was added to a Vero cell monolayer for 1 h at 37 °C. An overlay of 1.5% methylcellulose was added to the cells and followed by a 3 day incubation at 37 °C. The overlay was then removed, cells washed and fixed with a 1:1 methanol and acetone solution. The viral foci were stained using 4G2 antibody for 2 h followed by HRP conjugated anti-mouse IgG (Cell Signaling; 7076S) for 1 h and developed using TrueBlue peroxide substrate (KPL Seracare; Milford, MA, USA; 50-78-02). The foci were counted and analyzed to determine the dilution factor or concentration at which 50% reduction viral foci (FRNT50) was observed in the test samples compared to the virus only control.

### 2.10. Antibody Dependent Enhancement Assay

To assess the infection enhancement capacity of infected plasma and mAbs, an antibody dependent enhancement (ADE) assay was performed as previously described [13,33]. Briefly, serially diluted mAbs or heat inactivated plasma were incubated with 0.5 MOI of DENV2 for 1 h at 37 °C. The immune complexes were mixed with U937 cells (ATCC; Manassas, VA, USA; CRL-1593.2) in a 96-well plate for 24 h at 37 °C. The cells were then washed and intracellularly stained with 4G2 antibody using Fixation/Permeabilization solution kit (Becton Dickinson; Franklin Lakes, NJ, USA; 554714) followed by a secondary anti-mouse IgG AF488 (Life Technologies; Carlsbad, CA, USA; A11029). The percentage of infected (4G2+) cells was determined by flow cytometry using the LSR-II (Becton Dickinson; Franklin Lakes, NJ, USA) flow cytometer followed by analysis using FlowJo software v10.3.

## 3. Results

### 3.1. ZIKV Infection Induces Strong Recall Response in Flavivirus-Experienced Individual

**Figure 1:**
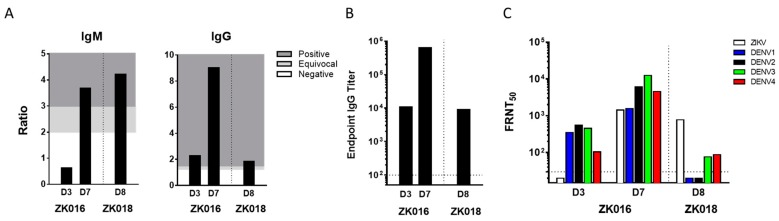
ZIKV neutralizing antibody titers detectable within days after ZIKV infection. (**A**) ZIKV-specific plasma IgM and IgG titers for patient ZK016 at 3 and 7 DPO and for ZK018 at 8 DPO. Titers were determined by ZIKV IgM antibody capture enzyme linked immunosorbent assay (MAC-ELISA) and modified IgG ELISA. The values are represented as ratios of the optical densities of patient plasma to healthy control plasma. For IgM, a ratio >3 = positive (dark gray), 2–3 = equivocal (light gray), >2 = negative (white) and for IgG, a ratio >1.5 = positive (dark gray), 1.2–1.5 = equivocal (light gray), >1.2 = negative (white). (**B**) Plasma endpoint IgG binding titers to ZIKV lysate as determined by ELISA. The dotted line indicates the starting plasma dilution of 1:100. (**C**) The Focus Reduction Neutralization Test (FRNT) titers of plasma from patients against ZIKV and DENV1-4. The values plotted are the plasma dilutions required for 50% neutralization of ZIKV foci, termed FRNT50. The FRNT assay for each sample was repeated in two or more independent experiments, and the mean values are plotted. The dotted line represents the starting plasma dilution of 1:30.

The two patients ZK016 and ZK018 included in this study were United States citizens who acquired ZIKV infections in the summer of 2016 during their residence (ZK016) or travels (ZK018) abroad. Details including patient demographics, timing of phlebotomies as days post onset of symptoms (DPO) and prior flavivirus history are summarized (Table 1). Plasma samples obtained at 3 and 7 DPO (ZK016), and 8 DPO (ZK018) were tested by ELISA and FRNT to determine ZIKV binding and neutralization titers (Figure 1). In the case of ZK016, ZIKV-specific IgM and IgG titers increased substantially between days 3 and 7, with higher anti-ZIKV IgG titers compared to IgM as early as 3 DPO (Figure 1A). In comparison, ZK018 appeared to have a delayed IgG response as higher ZIKV-specific IgM titers were observed at 8 DPO compared to anti-ZIKV IgG titers. Overall, ZK016 had markedly higher ZIKV-specific serum IgG than ZK018 (Figure 1B). As shown in Figure 1B, the day 7 IgG endpoint titer of ZK016 to ZIKV lysate was 100-fold greater than that of ZK018 at 8 DPO.

ZIKV-specific neutralizing antibodies were detected at 7 DPO and 8 DPO for patients ZK016 and ZK018 respectively. At 3 DPO, neutralization antibody titers were detected against all the 4 serotypes of DENV, but not against ZIKV in patient ZK016 despite seroconversion. In contrast, ZK018 plasma at 8 DPO did not neutralize DENV1 and DENV2, and showed low FRNT titers against DENV3 and DENV4 (Figure 1C). The early emergence of ZIKV-specific serum IgG, and comparable ZIKV and DENV neutralization titers taken together suggest that ZIKV infection primarily induced a recall response in patient ZK016. On the other hand, the high ZIKV-specific IgM levels, delayed emergence of ZIKV IgG and low cross-neutralizing DENV titers suggest that ZK018 may have mounted a primary response to ZIKV infection.

### 3.2. Robust Plasmablast Response Following ZIKV Infection

**Figure 2:**
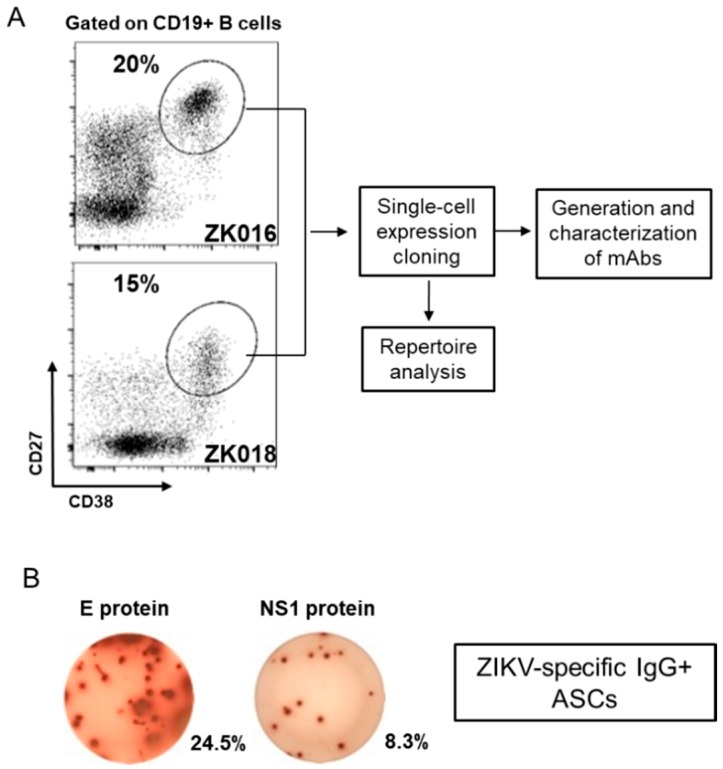
Virus-specific plasmablasts appear in circulation early after ZIKV infection. (**A**) Flow cytometry plots showing patient plasmablast (CD3− CD19+ CD20−/low CD38high CD27high) frequencies as a fraction of total CD19+ B cells. PBMCs were isolated from whole blood collected from patient ZK016 (7 DPO) and patient ZK018 (8 DPO) for flow cytometry analysis and plasmablast sorting. The workflow chart follows the generation and characterization of mAbs from single-cell sorted plasmablasts. (**B**) Representative ELISpot showing ZIKV E protein and NS1 protein-specific antibody secreting cells (ASCs). The wells shown contain 7 DPO PBMCs from patient ZK016. The percentages beside the wells represent the frequency of antigen-specific ASCs relative to the total number of IgG + ASCs.

At approximately one week after symptom onset, robust expansions of plasmablasts were apparent in circulation for both patients, amounting to 20% (ZK016) and 15% (ZK018) of their respective peripheral B cell populations (Figure 2A). As shown for ZK016 in Figure 2B, ZIKV E and NS1-specific, as well as ZIKV lysate-specific (data not shown) plasmablasts were observed at the DPO 7/8 time points for both patients by ELISpot. Patient plasmablasts were single-cell sorted to generate mAbs for B cell repertoire analysis and functional studies as outlined in the workflow chart (Figure 2A).

### 3.3. Recall Response Drives Expansion of Clonally-Linked, Cross-Reactive Plasmablasts

**Figure 3:**
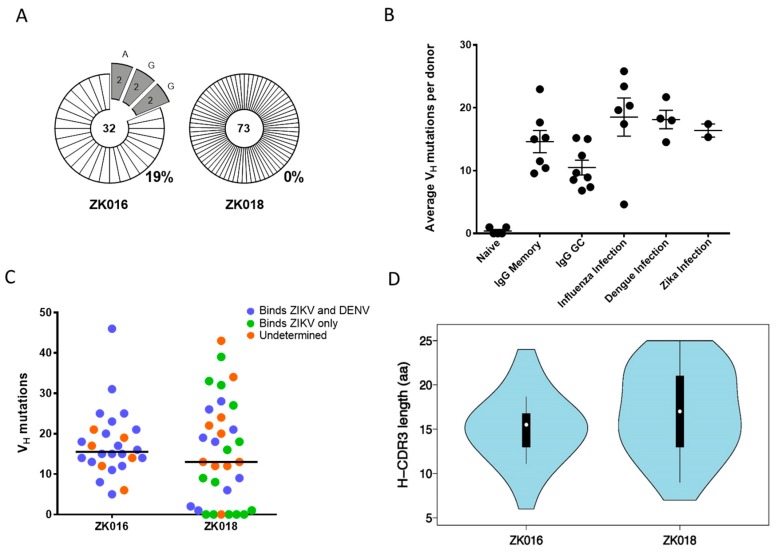
Clonality and mutational characteristics of the ZIKV plasmablast response. (**A**) Clonal relatedness of heavy chain variable region (VH) sequences amplified from patient plasmablasts. The number at the center of each pie chart is the total number of heavy chain sequences analyzed, including unpaired VH sequences that were not pursued for mAb synthesis. The numbers within the pie slices denote the number of clones with a shared VH rearrangement and junction. The percentage of total clonal sequences per donor is shown to the bottom right of the pie charts. (**B**) Average per donor VH mutation frequencies of the two ZIKV patients compared to historical data. Each circle represents the average number of VH nucleotide mutations per donor. Somatic hypermutation frequencies in naive, memory and germinal center B cells, and peripheral B cells from influenza and dengue infected donors were derived from previously published data [33,42]. (**C**) The range of VH mutation frequencies in the two ZIKV patients. Each circle represents the number of VH mutations per mAb sequence. The binding specificities of the mAbs to ZIKV and DENV are color coded: Cross-reactive mAbs are shown in blue, ZIKV-reactive in green and mAbs of undetermined specificity in orange. (**D**) VH CDR3 amino acid length distribution of all mAb sequences per patient.

During a recall response, memory B cells (MBCs) generated by a previous infection or vaccination can reactivate, resulting in expansions of plasmablasts that share a clonal origin. As shown in Figure 3A, 19% of the immunoglobulin sequences obtained from patient ZK016 were clonally related, whereas zero clonal expansions were detected in ZK018 plasmablasts. Analysis of VH somatic hypermutation (SHM) frequencies revealed no striking differences between patient ZK016 (average = 17.4; median = 15) and ZK018 (average = 15.3; median = 13). Nor were significant differences observed between the ZIKV patients (average = 16.4) compared to our previously reported SHM frequencies from dengue or influenza patient plasmablasts (Figure 3B) [33]. However, of note, 6 of the 33 mAbs generated from ZK018 plasmablasts were completely un-mutated and an additional 3 had 2 mutations or fewer in the VH region. In contrast, ZK016 plasmablasts SHM frequencies ranged from 5 to 46 per sequence, none of these mAbs were un-mutated (Figure 3C, Supplementary Table S1). A majority of mAbs with higher numbers of variable region mutations were cross-reactive to both ZIKV and DENV, while the 6 germline mAbs were found to be ZIKV-specific (Figure 3C, Supplementary Table S1). We also analyzed heavy chain CDR3 lengths and found that patient ZK016 plasmablasts had a normal distribution with 5 sequences representing the peak CDR3 length of 16 aa (Figure 3D). On the other hand, ZK018 CDR3 lengths more closely resembled a multimodal distribution, with 3 sequences each at the 13 aa, 14 aa and 23 aa modes (Figure 3D).

### 3.4. Primary and Recall ZIKV Plasmablast Responses Differ in Frequency and Breadth of Cross-Reactivity.

**Figure 4:**
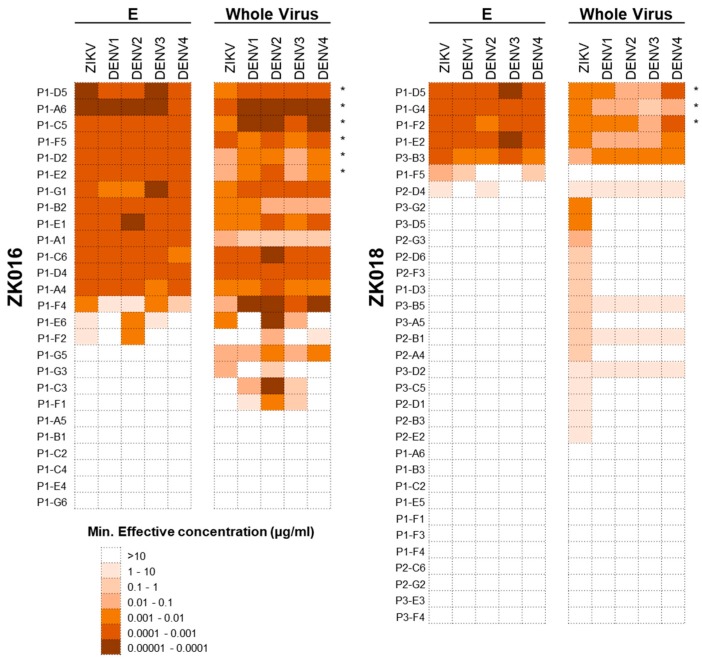
The recall ZIKV plasmablast response is highly cross-reactive against DENV. Heat map showing the binding potency of the ZK016 and ZK018 mAbs against ZIKV and DENV. The mAbs generated from single-cell sorted plasmablasts were tested for reactivity against ZIKV and DENV1-4 recombinant E protein (E) and whole virion by ELISA. The mAbs that are fusion loop (FL)-specific are indicated with asterisks. In terms of scale, brown represents the highest and white the lowest potency of binding based on minimum effective concentration. The minimum effective concentration is the minimum mAb concentration required to obtain three times the background signal. The results plotted are mean values from two or more independent ELISA experiments.

To dissect the plasmablast response induced by ZIKV infection, we generated a panel of 59 mAbs from the two patients combined: 26 mAbs from ZK016 (derived from 18 IgG and 8 IgA plasmablasts) and 33 mAbs from ZK018 (derived from 23 IgG and 10 IgA plasmablasts). Of the 26 mAbs generated from ZK016, 20 (77%) bound to ZIKV E and/or whole virus. All ZK016 mAbs that bound ZIKV antigens also cross-reacted with DENV, largely with equal or greater potency (Figure 4). By contrast, a smaller frequency of ZK018 mAbs, 22 of the 33 mAb total (67%) were ZIKV E or whole virus-reactive, of which 6 exhibited poor binding (minimum effective concentrations = 1–10 µg/mL) to these antigens. Additionally, only 9 mAbs (27%) from ZK018 were cross-reactive to DENV. A majority of cross-reactive mAbs from both patients were mapped to the highly conserved fusion loop (FL) (Supplementary Figure S2). Taken together, these data show that the primary response generated by ZK018 comprised non-affinity matured, de novo plasmablasts that were largely weakly-reactive and ZIKV-specific. In contrast, for ZK016 the selection and reactivation of pre-existing cross-reactive MBCs may have resulted in a predominantly high affinity, cross-reactive plasmablast repertoire.

### 3.5. Neutralization Bias towards DENV Observed in ZIKV Plasmablast Response of Dengue-Experienced Patient

**Figure 5:**
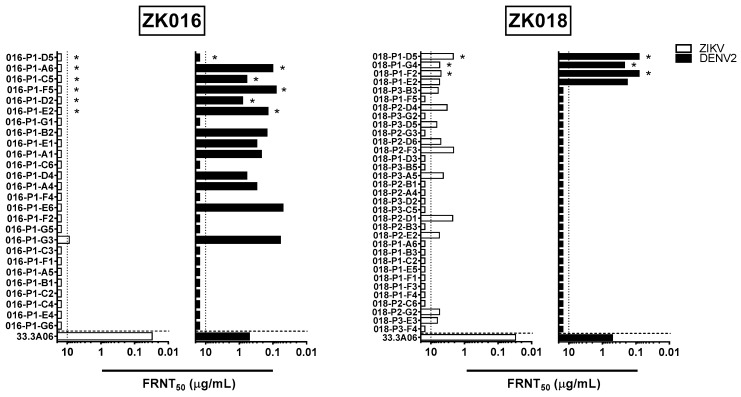
Plasmablast-derived mAbs from dengue-experienced individual preferentially neutralize DENV compared to ZIKV. The mAbs generated from patient ZK016 (**left** panels) and ZK018 (**right** panels) were tested for neutralization activity against ZIKV (white bar) and DENV2 (black bar). FL-specific mAbs are indicated by asterisks. The values plotted represent mAb concentration required for 50% reduction of viral foci, termed FRNT50. The vertical dotted lines indicate the maximum concentrations of mAb tested (10 μg/mL). The horizontal dotted line separates the positive control, dengue plasmablast-derived mAb 33.3A06 reported previously [33]. The results plotted are mean values from two or more independent experiments.

All ZK016 and ZK018 mAbs were tested against ZIKV and DENV1-4 for in vitro neutralization capacity (Figure 5, Supplementary Figure S1). For ZK016, only one mAb sufficiently neutralized ZIKV to yield an FRNT50 value within the range of concentrations tested. This mAb, P1-G3, poorly neutralized ZIKV with an FRNT50 value of 8.74 µg/mL. Interestingly, despite the limited ZIKV neutralization capacity of ZK016 mAbs, an additional 11 mAbs besides P1-G3 from this donor cross-neutralized DENV2. These mAbs neutralized DENV with >10-fold greater potency, with FRNT50 values ranging between 50 and 800 ng/mL. This neutralization bias towards DENV was not observed in mAbs from ZK018; 14 of the 33 mAbs neutralized ZIKV, and only 4 of these ZIKV-neutralizers also neutralized DENV. ZK018 mAbs neutralized ZIKV at comparable or slightly higher potency than P1-G3, with FRNT50 values ranging from 2.1 to 6.66 µg/mL. The 4 mAbs that neutralized DENV1-4 were all FL-specific (Figure 5, Supplementary Figure S1). These findings suggest that a number of DENV-biased, ZIKV-reactive B cell clones were selected for reactivation during the ZK016 recall response to ZIKV, resulting in an original antigenic sin (OAS) neutralization phenotype. In the absence of pre-existing dengue immunity, ZK018 generated a de novo plasmablast response selected primarily on the basis of reactivity towards ZIKV. Therefore, a greater number of ZK018 clones neutralized ZIKV overall, including a handful that also cross-neutralized DENV.

### 3.6. Potent Antibody-Dependent Enhancement of ZIKV by ZK016 mAbs

**Figure 6:**
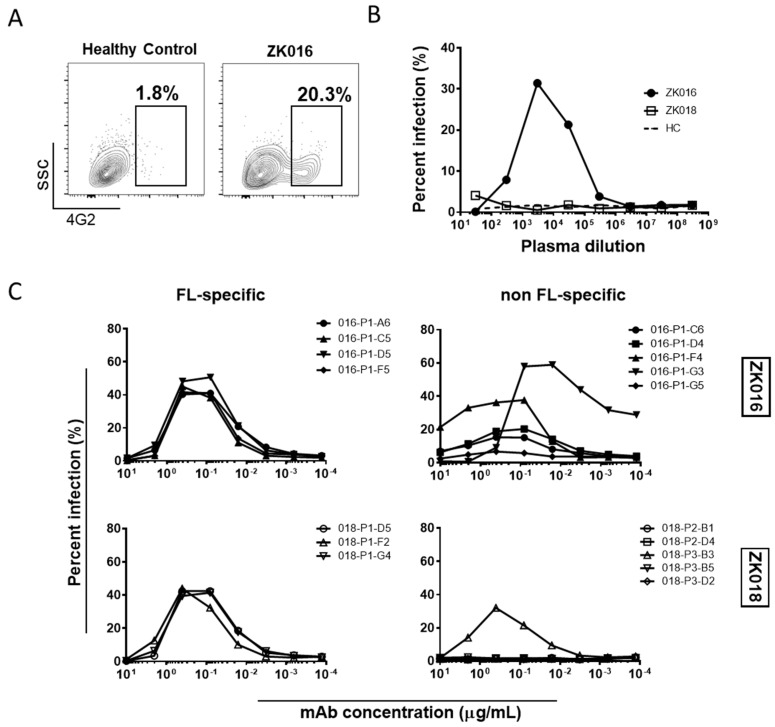
ZIKV plasmablast responses enhance DENV2 infection in vitro. (**A**) Representative flow cytometry plots comparing ADE activity of plasma from ZK016 vs. healthy control. Boxed population represents DENV2-infected cells based on positive 4G2 staining. Plots shown display ADE at plasma dilution of 1:3000. (**B**) DENV2 ADE activities of plasma from ZK016, ZK018 and a flavivirus-naïve healthy donor (dotted line). (**C**) DENV2 ADE activities of FL-specific and non FL-specific mAbs from ZK016 and ZK018.

ADE is one of several hypothesized causes for the increased disease severity associated with secondary heterotypic dengue infections [43]. Recently, several groups have studied ADE in the context of ZIKV infection in efforts to understand whether pre-existing cross-reactive antibodies from a prior DENV exposure can affect ZIKV infection, and vice-versa [13,23,44,45]. Given that both patients in our study had recently experienced ZIKV infections, we first examined whether plasma collected early after ZIKV exposure could enhance DENV2 infection through ADE. We tested ADE using an Fc-gamma receptor-bearing monocytic cell line, U937, and determined percent infection in the presence or absence of antibodies based on 4G2+ staining (Figure 6A). As shown in Figure 6B, 7 DPO plasma from ZK016 caused considerable ADE of DENV2, infecting over 30% of cells at the peak ADE titer. By contrast, 8 DPO plasma from patient ZK018 did not enhance DENV2 infection. To determine which types of B cell clones were contributing to the plasma ADE titers, we tested the ability of select mAbs from both patients to cause ADE. As expected, mAbs that strongly bound ZIKV E and whole virus caused ADE of DENV2, while mAbs that showed little to no reactivity to these antigens largely failed to enhance infection (Figure 6C). In general, FL-specific mAbs caused greater enhancement of DENV2 infection compared to non FL-specific mAbs, and a larger number of mAbs from ZK016 enhanced DENV2 infection compared to ZK018.

### 3.7. NS1 Protein Targeted by Type-Specific and Cross-Reactive ZIKV Plasmablasts

**Figure 7:**
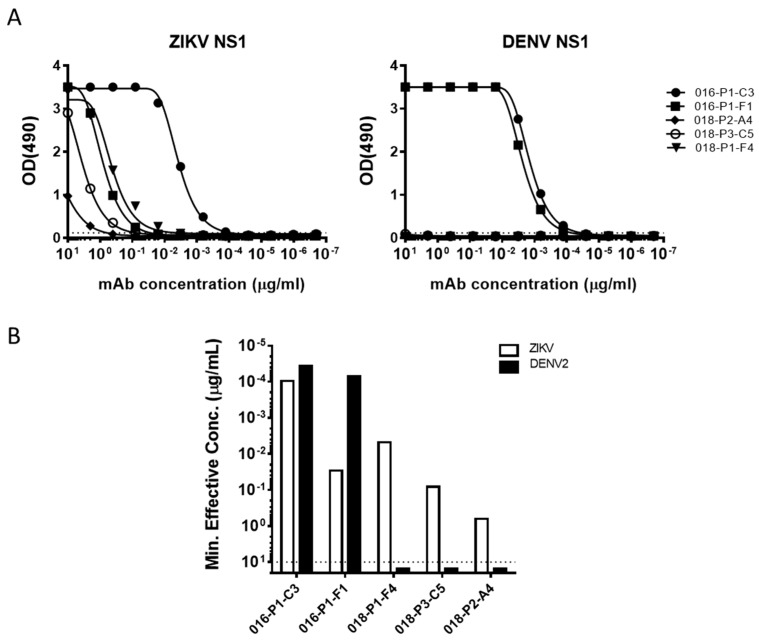
ZIKV infection drives activation of NS1-specific plasmablast responses. (**A**) Binding curves of ZK016 and ZK018 NS1-specific mAbs. Antibodies were tested for binding to ZIKV and DENV2 recombinant NS1 proteins by ELISA. The dotted lines represent three times the background signal obtained with plain blocking buffer. (**B**) Binding activity of mAbs to ZIKV (white bar) or DENV2 (black bar) recombinant NS1 protein by ELISA. The values plotted represent the minimum concentrations required to obtain three times the background signal. The dotted line indicates the maximum concentration of mAbs tested in ELISA (10 μg/mL). Results plotted are representative of two or more independent ELISA experiments.

While a majority of mAbs generated from the two donors were either E-protein or whole virus-reactive, we also observed a few that bound NS1 protein. Of the 5 ZIKV NS1-specifc mAbs, two came from ZK016 and three from ZK018 (Figure 7A). Both ZK016 mAbs cross-reacted to ZIKV and DENV NS1 proteins, binding DENV NS1 at equal or higher potency compared to ZIKV NS1 (Figure 7B). Surprisingly, ZK018 NS1 mAbs were ZIKV-specific and did not bind DENV NS1, even though NS1 is known to be highly conserved in flaviviruses (Figure 7). Additionally, the ZIKV NS1-specific mAbs from ZK018 had little to no SHM (Supplementary Table S1). Given the difficulties in discerning ZIKV infections from DENV based on serology, these ZIKV-NS1 specific antibodies could be pursued as potential diagnostic tools for use early after ZIKV infection.

## 4. Discussion

In this study, we analyzed the acute phase B cell response in two ZIKV-infected patients by functionally characterizing mAbs derived from single-cell sorted donor plasmablasts. One of the two patients in this study was previously infected with DENV (ZK016), while the other likely had no prior DENV exposures (ZK018) at the time of sample collection. In both donors, substantial expansions of plasmablasts in peripheral blood, and considerable ZIKV-specific binding and neutralization plasma titers were detected within a week after symptom onset. Based on the abundance of ZIKV-specific IgG, and high DENV1-4 cross-neutralizing plasma antibody titers, we hypothesized that ZK016 was generating a recall response to ZIKV. The presence of high ZIKV-specific IgM binding titers, and low plasma cross-neutralization capacity against DENV1-4 suggested that ZK018 was experiencing a primary response to ZIKV infection. The detection of several clonal expansions in the ZK016 plasmablast population, and the simultaneous absence of any clonal relatedness between ZK018 plasmablasts, supported our classification of ZK016 and ZK018 as recall and primary responders, respectively.

Cryo-EM reconstructions of mature ZIKV particles, super-impositions of ZIKV and DENV E proteins and viral sequence alignments have altogether revealed a great degree of structural similarity and sequence homology in immunodominant sites between the two viruses [12,13,46,47]. Studies of mAbs derived from acute or convalescent DENV-infected individuals have shown high ZIKV E-directed cross-reactivity [13,19,20,23]. Conversely, mAbs generated from MBCs of ZIKV-infected DENV-naïve or immune individuals also exhibit cross-reactivity to the DENV E protein [20]. Although DENV-ZIKV immunological cross-reactivity has been widely documented and reported, the factors that modulate this cross-reactivity are less well understood. In order to determine whether prior dengue immunity can impact the breadth and potency of ZIKV-specific vs. cross-reactive antibody responses, we tested each of the 59 mAbs generated from patient plasmablasts against ZIKV and DENV1-4 for binding and neutralization. All ZIKV-specific mAbs from the dengue-experienced ZK016 cross-reacted to DENV1-4 antigens. About one-third of these mAbs were mapped to the fusion loop, which is located within DII of the E protein and is highly conserved between flaviviruses [48]. Interestingly, a majority of the cross-reactive mAbs preferentially neutralized DENV1-4 over ZIKV, which was highly reminiscent of OAS. This OAS phenotype of recall response mAbs was also observed by Rogers et al., who showed a neutralization bias towards DENV in the plasmablast response of three dengue-experienced donors [21]. While several mAbs in the Rogers et al. study neutralized ZIKV in addition to DENV, in our study, all but one mAb, P1-G3, failed to neutralize ZIKV even at the maximal concentration tested (20 µg/mL). This singular ZIKV-neutralizing mAb did not bind recombinant ZIKV E protein but did bind whole ZIKV virions, suggesting that it may target a quaternary epitope that is dependent on E protein conformation.

Several such non-E protein-binding but ZIKV virion-reactive mAbs were identified in the naïve responder ZK018. Of the 14 mAbs exhibiting this phenotype, only 3 cross-reacted to DENV1-4 whereas all E-binding mAbs cross-reacted to DENV1-4 E proteins. Therefore, fewer mAbs from the dengue-naive donor were ZIKV-reactive compared to ZK016, a majority of which were ZIKV virion-specific and did not cross-react to DENV, similar to the findings of Rogers et al. [21]. Despite lower ZIKV-reactivity overall, a larger number of ZK018 mAbs neutralized ZIKV compared to ZK016, albeit with low to moderate neutralization potency. The subpar neutralization capacity of the mAbs towards ZIKV could potentially be due to limited epitope accessibility on the viral surface. Kostyuchenko et al. reported that the ZIKV surface is more compact and the virus more thermally stable than DENV2 [49]. These differences may affect the extent of “virus breathing”, which could be an important determinant for epitope accessibility and recognition [50].

Besides their potential role in protection against heterologous infections, cross-reactive antibodies have also been implicated in exacerbating disease severity through ADE [45,51]. We tested whether plasma or mAbs from the two ZIKV-infected patients in our study enhanced DENV2 infection using FcRγ-expressing U937 cells. While ZK016 potently enhanced DENV2 infection, minimal infection of U937 cells was observed in the presence of ZK018 plasma. The minimal DENV2 enhancing activity of ZK018 plasma mirrors the findings of Ricciardi et al., who reported a lack of DENV2 enhancing activity in the plasma of a dengue-naïve ZIKV-infected individual for as long as 150 DPO [32]. This vast difference in the ADE capacities of ZK016 and ZK018 plasma could be attributed to the quantities DENV cross-reactive antibodies in plasma. We hypothesized that the antibody threshold required for ADE of DENV2 infection was not met in ZK018 due to low prevalence of DENV-reactive antibodies. To dissect the polyclonal responses of the two patients, we determined the ADE capacities of a subset of mAbs from each donor. In general, FL-specific mAbs from both donors enhanced DENV2 to a greater extent than non FL-specific mAbs. In case of ZK016, several E-binding non FL-specific mAbs also enhanced DENV2 infection, whereas for ZK018, DENV2 enhancing activity was largely restricted to FL-specific mAbs. These FL-specific, DENV2 enhancing antibodies could be the cause for the low levels of ADE observed at the plasma level for ZK018. Overall, our results suggest that circulating antibodies immediately following a ZIKV infection have a lower propensity for enhancing DENV infection in an individual without prior dengue exposures. Further, the possibility for ADE during sequential ZIKV-DENV infections may be enhanced by the presence of pre-existing DENV antibodies.

In addition to the E-protein specific and whole virus-reactive mAbs, we identified five mAbs that were specific to the NS1 protein. Interestingly, the two NS1-specific mAbs generated from ZK016 were cross-reactive to both DENV and ZIKV NS1 protein, while three NS1-specific mAbs generated from ZK018 exclusively bound ZIKV NS1 protein. The cross-reactive phenotype of ZK016 NS1 mAbs and lack of DENV binding by ZK018 NS1 mAbs echoes the E protein binding patterns of mAbs from the two donors. These findings suggest that in addition to impacting the E-specific B cell repertoire, the NS1-specific response after ZIKV infection can also be affected by pre-existing dengue immunity. Recent reports have demonstrated significant differences between the electrostatic potentials of the loop-surface interface of DENV and ZIKV NS1 [52,53,54]. These differences could affect antibody recognition and binding, and may explain the type-specific binding observed with ZK018 mAbs despite the high sequence identity (51–55%) between ZIKV and DENV NS1 proteins reported previously [20,52]. Since NS1 is the only viral protein that is secreted from infected cells, its early detection after infection can be an important diagnostic tool in resource-limited settings where viral detection or typing are challenging. Given that the three ZK018 NS1 mAbs only bind ZIKV and not DENV, these mAbs have immense clinical potential as a diagnostic tool to differentiate between ZIKV and DENV infections. We are currently evaluating the diagnostic potential of these mAbs in ongoing studies.

The two donors in our study differed in immune status at the time of their ZIKV exposure: while one had previously been exposed to DENV, the other appeared to be DENV-naïve at the time of ZIKV infection. The resulting plasmablast response differed in levels of cross-reactivity, and in the binding, neutralization and infection-enhancing phenotypes of antibodies. Although our study is limited in the number of patients analyzed, our data are strongly supported by the findings of Rogers et al. who performed a longitudinal analysis of the B cell response in three dengue-experienced and one dengue-naïve individual. Together, our observations demonstrate that pre-existing dengue immunity may promote an OAS-like DENV bias in the ZIKV recall response. In addition, ZIKV infection may enhance the breadth and potency of antibody reactivity towards DENV in a dengue-experienced individual, while de novo plasmablast responses remain largely ZIKV-specific. Given that future ZIKV epidemics may affect populations with varied dengue serostatus, these findings emphasize the importance of evaluating ZIKV vaccine efficacy in both dengue-naïve and dengue-immune individuals.

## Figures and Tables

**Table 1:** Patient information.

Patient	Age/Sex	Region of ZIKV	Plasmablasts Analyzed	Previous Dengue
		Exposure	(DPO *)	Exposure **
ZK016	32/Female	Honduras	3, 7	Yes
ZK018	26/Female	Caribbean Islands	8	No

* DPO-Days post onset of symptoms; ** Based on DENV plasma Ab levels.

## Supplementary Materials

The following are available online at http://www.mdpi.com/1999-4915/11/1/19/s1, Figure S1: ZK016 plasmablast-derived mAbs exhibit wider cross-neutralization capacity against DENV compared to ZK018 mAbs, Figure S2: Epitope mapping of fusion loop-specific antibodies by competition ELISA; Table S1: Repertoire and mutational analysis of ZK016 and ZK018 mAbs.
