# Supplementary material for: Pre-Existing Dengue Immunity Drives a DENV-Biased Plasmablast Response in ZIKV-Infected Patient
Source: Viruses. 2018 Dec 29;11(1):19. doi: 10.3390/v11010019 (PMC6356269; doi:10.3390/v11010019)
Supplement: Supplementary file 1 [file viruses-11-00019-s001.pdf]

Supplementary Material

Table S1. Repertoire and mutational analysis of ZK016 and ZK018 mAbs.

Patient	Mab	Heavy Chain			CDR3 length	No.of mutations		Light Chain			CDR3 length	No. of mutations	
		Original Ig Isotype*	V gene	J gene		Total	S/R	Kappa/Lambda	V gene	J gene		Total	S/R
**ZK016**	P1-A1	IgA1	1-24	4	6	5	2/3	Igκ	1-13	5	9	6	1/5
	P1-A4	IgG1	4-39	5	21	17	3/14	Igκ	3D-20	5	9	4	1/3
	P1-A5	IgA1	5-10	4	13	12	0/12	Igλ	1-44	2	10	13	2/11
	P1-A6	IgG1	4-39	4	18	31	8/23	Igκ	3-15	1	11	9	2/7
	P1-B1	IgA1	3-64	4	16	14	3/11	Igκ	1D-13	5	9	5	1/4
	P1-B2	IgG1	3-21	3	12	15	4/11	Igλ	3-21	3	11	12	3/9
	P1-C2	IgA2	3-74	4	10	19	7/12	Igλ	2-8	3	10	6	1/5
	P1-C3	IgG1	3-48	6	16	15	2/13	Igκ	3-20	2	10	5	1/4
	P1-C4	IgA1	3-64	4	16	17	3/14	Igκ	1D-13	5	9	6	3/3
	P1-C5	IgG1	1-18	5	19	14	0/14	Igλ	1-44	2	11	10	4/6
	P1-C6	IgG1	1-69	1	17	23	8/15	Igκ	1-39	2	9	15	5/10
	P1-D2	IgG1	3-30	4	18	11	2/9	Igλ	1-40	2	11	7	0/7
	P1-D4	IgG1	3-30	4	13	21	4/17	Igκ	1-27	4	9	18	6/12
	P1-D5	IgG1	3-15	4	9	12	4/8	Igλ	2-14	1	10	12	2/10
	P1-E1	IgG1	1-69	6	16	20	7/13	Igλ	2-11	3	10	20	4/16
	P1-E2	IgG1	4-39	5	13	25	7/18	Igκ	1-9	5	9	9	1/8
	P1-E4	IgA1	3-21	4	14	21	8/13	Igλ	3-25	2	11	21	7/14
	P1-E6	IgG1	4-39	3	15	16	8/8	Igλ	1-44	3	11	12	1/11
	P1-F1	IgG1	3-48	6	12	18	4/14	Igκ	3-20	2	10	11	2/9
	P1-F2	IgG1	1-2	6	21	14	3/11	Igλ	3-25	2	11	15	2/13
	P1-F4	IgG1	3-13	4	11	15	4/11	Igλ	2-14	3	10	16	3/13
	P1-F5	IgA1	4-38	4	14	25	9/16	Igκ	3-20	4	8	14	3/11
	P1-G1	IgG1	1-18	5	16	13	4/9	Igλ	2-23	2	10	12	4/8
	P1-G3	IgG1	3-23	6	24	8	1/7	Igλ	2-14	2	10	4	0/4
	P1-G5	IgG1	4-39	4	15	46	0/46	Igλ	1-51	2	11	7	4/3
	P1-G6	IgA1	3-21	4	16	6	2/4	Igλ	1-51	2	11	7	1/6
**ZK018**	P1-A6	IgG1	4-39	3	16	34	8/26	Igκ	1-39	1	9	25	11/14
	P1-B3	IgG1	3-64	6	19	12	2/10	Igλ	1-40	3	11	9	1/8
	P1-C2	IgG1	3-23	5	17	20	5/15	Igκ	3-15	1	8	12	5/7
	P1-D3	IgA1	3-30	6	22	8	3/5	Igλ	1-44	2	11	13	1/12
	P1-D5	IgG1	3-23	4	21	21	3/18	Igκ	3-20	5	8	13	2/11
	P1-E2	IgG1	3-43	6	23	9	2/7	Igκ	2D-29	5	9	8	0/8
	P1-E5	IgA2	3-48	4	18	12	2/10	Igκ	1-6	2	9	4	4/0
	P1-F1	IgG1	3-9	6	23	13	5/8	Igλ	1-44	3	12	4	2/2
	P1-F2	IgA1	4-39	5	10	6	2/4	Igκ	1-9	3	9	2	0/2
	P1-F3	IgA1	4-31	4	10	43	12/31	Igλ	7-46	3	10	24	5/19
	P1-F4	IgG1	3-66	3	9	0	0/0	Igκ	1-39	2	9	0	0/0
	P1-F5	IgG2	3-30	4	15	39	6/33	Igκ	1-5	5	9	23	9/14
	P1-G4	IgG1	4-39	4	11	19	4/15	Igλ	1-40	1	11	5	0/5
	P2-A4	IgG1	4-59	3	14	0	0/0	Igκ	1-5	4	9	0	0/0
	P2-B1	IgG2	1-46	4	16	26	4/22	Igκ	1-5	2	10	20	4/16
	P2-B3	IgG1	3-33	4	13	0	0/0	Igλ	8-61	3	10	0	0/0
	P2-C6	IgA2	1-46	4	12	13	1/12	Igκ	3-20	2	11	7	3/4
	P2-D1	IgA1	4-4	6	14	0	0/0	Igκ	1-39	2	9	0	0/0
	P2-D4	IgG1	1-69	6	20	2	1/1	Igλ	3-1	2	9	0	0/0
	P2-D6	IgA1	4-39	6	21	33	5/28	Igκ	1-27	1	10	15	8/7
	P2-E2	IgG2	1-18	3	13	18	4/14	Igλ	4-69	3	9	19	5/14
	P2-F3	IgA1	3-30	4	23	27	3/24	Igκ	3D-15	4	10	12	2/10
	P2-G2	IgA1	5-51	4	16	24	5/19	Igκ	3-15	2	11	18	5/13
	P2-G3	IgA1	4-34	5	22	32	8/24	Igλ	3-19	3	11	16	4/12
	P3-A5	IgG1	6-1	5	24	16	5/11	Igκ	3-20	5	9	6	2/4
	P3-B3	IgG1	3-30	6	17	18	8/10	Igκ	2-24	2	8	6	4/2
	P3-B5	IgG2	3-23	4	13	28	4/24	Igκ	3-11	5	10	15	3/12
	P3-C5	IgG1	3-53	4	7	1	1/0	Igκ	1-39	2	9	0	0/0
	P3-D2	IgG1	1-69	6	25	1	0/1	Igλ	3-1	2	9	0	0/0
	P3-D5	IgG1	3-7	6	20	0	0/0	Igκ	3-15	5	10	0	0/0
	P3-E3	IgG2	4-61	4	14	22	6/16	Igκ	4-1	2	9	39	6/33
	P3-F4	IgG1	3-43	6	24	0	0/0	Igλ	3-21	2	11	1	1/0
	P3-G2	IgG1	4-31	4	18	9	1/8	Igλ	3-1	2	9	16	3/13

*All mAbs were cloned into IgG heavy chain expression vector.
Clonal mAbs shaded in gray.

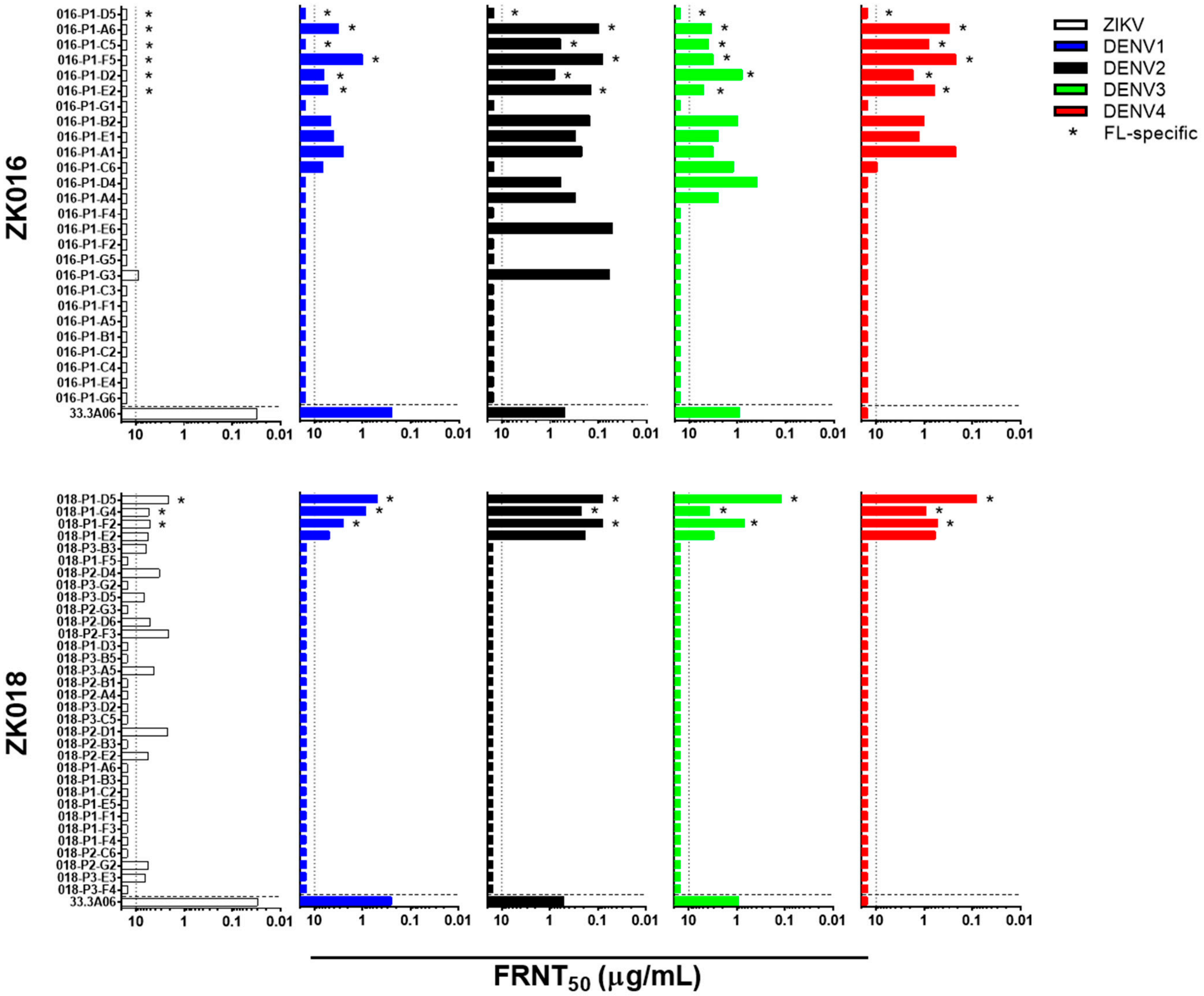

Figure S1. ZK016 plasmablast-derived mAbs exhibit wider cross-neutralization capacity against DENV compared to ZK018 mAbs. All mAbs were tested for neutralization activity against ZIKV (white), DENV1 (blue), DENV2 (black), DENV3 (green) and DENV4 (red). Fusion-loop (FL)-specific mAbs are indicated by asterisks. The values plotted represent mAb concentrations required for 50% reduction of viral foci (FRNT50). The vertical dotted lines indicate the maximum concentration of mAbs tested (10 µg/mL). The horizontal dotted lines separate 33.3A06, a dengue plasmablast-derived mAb reported previously [33]. The values plotted represent the mean of two or more independent FRNT experiments.

A

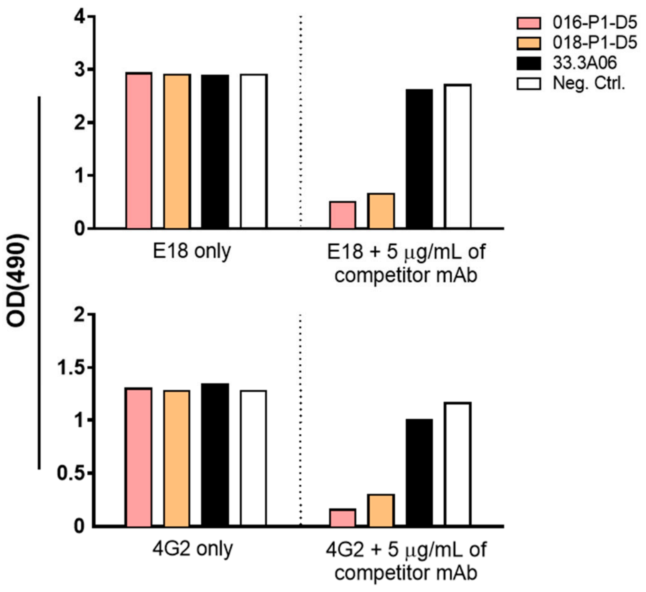

B

Competitor mAb	Percent Reduction in Binding	
	E18	4G2
016-P1-A6	75.7	74.7
016-P1-D5	73.7	73.0
016-P1-F5	63.7	64.9
016-P1-D2	63.5	68.4
016-P1-E2	54.7	56.4
016-P1-C5	42.6	50.5
016-P1-G1	35.6	46.3
016-P1-B2	24.2	31.2
016-P1-A1	-1.4	16.9
016-P1-F4	-5.3	11.2
016-P1-F2	-15.9	-2.1
016-P1-C6	-16.9	3.9
016-P1-E6	-18.1	7.7
016-P1-A4	-20.6	4.5
016-P1-D4	-23.9	-4.0
016-P1-E1	-30.9	-4.8
018-P1-G4	60.3	62.1
018-P1-D5	59.8	59.8
018-P1-F2	52.8	55.5
018-P1-E2	40.1	48.6
018-P3-B3	12.6	26.2
018-P1-F5	-15.3	3.7
33.3A06	-1.4	9.0
Neg. Ctrl.	-13.8	6.5

Figure S2. Epitope mapping of fusion loop-specific antibodies by competition ELISA. (A) Reduction in the binding of E18 (top panel) and 4G2 (bottom panel), two murine FL-specific mAbs, to ZIKV rE in the presence of patient mAbs. One mAb each from ZK016 and ZK018 are shown as representative examples of significant reduction in ELISA binding signal. Also shown are competition activities of two mAbs that do not bind ZIKV FL: 33.3A06 [33] and a negative control V. cholera LPS-specific mAb. (B) Percent reduction in binding of E18 and 4G2 to ZIKV rE in the presence of ZK016 and ZK018 mAbs. All mAbs that caused >50% reduction in binding were deemed FL-specific and are shaded blue.
